# Supplementary material for: Pyruvate Dehydrogenase Kinase 1 inhibition mediated oxidative phosphorylation enhancement in cartilage promotes osteoarthritis progression
Source: BMC Musculoskelet Disord. 2023 Jul 20;24:597. doi: 10.1186/s12891-023-06585-6 (PMC10357736; doi:10.1186/s12891-023-06585-6)
Supplement: Supplementary file 1 — Additional file 1. [file 12891_2023_6585_MOESM1_ESM.zip › MF.Gsea.1653627316242/GOMF_CYCLOSPORIN_A_BINDING.html]

Details for gene set GOMF\_CYCLOSPORIN\_A\_BINDING[GSEA]

|  || Dataset | 55457\_collapsed\_to\_symbols.cls.cls #OA\_versus\_normal.cls.cls #OA\_versus\_normal\_repos |
| Phenotype | cls.cls#OA\_versus\_normal\_repos |
| Upregulated in class | normal |
| GeneSet | GOMF\_CYCLOSPORIN\_A\_BINDING |
| Enrichment Score (ES) | -0.5076504 |
| Normalized Enrichment Score (NES) | -1.4763749 |
| Nominal p-value | 0.060606062 |
| FDR q-value | 0.20381896 |
| FWER p-Value | 1.0 |
Table: GSEA Results Summary

  

Fig 1: Enrichment plot: GOMF\_CYCLOSPORIN\_A\_BINDING      
 Profile of the Running ES Score & Positions of GeneSet Members on the Rank Ordered List

  

| SYMBOL | TITLE | RANK IN GENE LIST | RANK METRIC SCORE | RUNNING ES | CORE ENRICHMENT || 1 | PPIE | peptidylprolyl isomerase E [Source:HGNC Symbol;Acc:HGNC:9258] | 334 | 0.733 | 0.0777 | No |
| 2 | PPIH | peptidylprolyl isomerase H [Source:HGNC Symbol;Acc:HGNC:14651] | 1555 | 0.419 | 0.0423 | No |
| 3 | PPIC | peptidylprolyl isomerase C [Source:HGNC Symbol;Acc:HGNC:9256] | 1823 | 0.381 | 0.0755 | No |
| 4 | PPIB | peptidylprolyl isomerase B [Source:HGNC Symbol;Acc:HGNC:9255] | 3372 | 0.214 | -0.0144 | No |
| 5 | PPP3CA | protein phosphatase 3 catalytic subunit alpha [Source:HGNC Symbol;Acc:HGNC:9314] | 3803 | 0.176 | -0.0229 | No |
| 6 | PPID | peptidylprolyl isomerase D [Source:HGNC Symbol;Acc:HGNC:9257] | 5029 | 0.071 | -0.1080 | No |
| 7 | PPIAL4G | peptidylprolyl isomerase A like 4G [Source:HGNC Symbol;Acc:HGNC:33996] | 6779 | -0.061 | -0.2351 | No |
| 8 | PPWD1 | peptidylprolyl isomerase domain and WD repeat containing 1 [Source:HGNC Symbol;Acc:HGNC:28954] | 9069 | -0.237 | -0.3792 | No |
| 9 | PPIL2 | peptidylprolyl isomerase like 2 [Source:HGNC Symbol;Acc:HGNC:9261] | 9148 | -0.244 | -0.3507 | No |
| 10 | PPIF | peptidylprolyl isomerase F [Source:HGNC Symbol;Acc:HGNC:9259] | 9999 | -0.321 | -0.3714 | No |
| 11 | PPIL6 | peptidylprolyl isomerase like 6 [Source:HGNC Symbol;Acc:HGNC:21557] | 11756 | -0.541 | -0.4311 | Yes |
| 12 | PPIA | peptidylprolyl isomerase A [Source:HGNC Symbol;Acc:HGNC:9253] | 11986 | -0.591 | -0.3654 | Yes |
| 13 | PPIG | peptidylprolyl isomerase G [Source:HGNC Symbol;Acc:HGNC:14650] | 12358 | -0.695 | -0.2960 | Yes |
| 14 | NKTR | natural killer cell triggering receptor [Source:HGNC Symbol;Acc:HGNC:7833] | 12518 | -0.757 | -0.2013 | Yes |
| 15 | RANBP2 | RAN binding protein 2 [Source:HGNC Symbol;Acc:HGNC:9848] | 12609 | -0.804 | -0.0947 | Yes |
| 16 | PPP3R1 | "protein phosphatase 3 regulatory subunit B, alpha [Source:HGNC Symbol;Acc:HGNC:9317]" | 12653 | -0.829 | 0.0192 | Yes |
Table: GSEA details [plain text format]

  

Fig 2: GOMF\_CYCLOSPORIN\_A\_BINDING      
 Blue-Pink O' Gram in the Space of the Analyzed GeneSet

  

Fig 3: GOMF\_CYCLOSPORIN\_A\_BINDING: Random ES distribution      
 Gene set null distribution of ES for **GOMF\_CYCLOSPORIN\_A\_BINDING**
